# SF-CNN: Signal Filtering Convolutional Neural Network for Precipitation Intensity Estimation

**DOI:** 10.3390/s22020551

**Published:** 2022-01-11

**Authors:** Chih-Wei Lin, Xiuping Huang, Mengxiang Lin, Sidi Hong

**Affiliations:** 1 College of Computer and Information Science, Fujian Agriculture and Forestry University, Fuzhou 350002, China; hxp@fafu.edu.cn (X.H.); lmx@fafu.edu.cn (M.L.); 2 College of Forestry, Fujian Agriculture and Forestry University, Fuzhou 350002, China; 3 Forestry Post-Doctoral Station, Fujian Agriculture and Forestry University, Fuzhou 350002, China; 4 Key Laboratory of Fujian Universities for Ecology and Resource Statistics, Fujian Agriculture and Forestry University, Fuzhou 350002, China; 5 Cross-Strait Nature Reserve Research Center, Fujian Agriculture and Forestry University, Fuzhou 350002, China; 6 College of New Engineering Industry, Putian University, Putian 351100, China; hongsidi@ptu.edu.cn

**Keywords:** precipitation intensity, signal filtering, dimensional reduction

## Abstract

Precipitation intensity estimation is a critical issue in the analysis of weather conditions. Most existing approaches focus on building complex models to extract rain streaks. However, an efficient approach to estimate the precipitation intensity from surveillance cameras is still challenging. This study proposes a convolutional neural network known as the signal filtering convolutional neural network (SF-CNN) to handle precipitation intensity using surveillance-based images. The SF-CNN has two main blocks, the signal filtering block (SF block) and the gradually decreasing dimension block (GDD block), to extract features for the precipitation intensity estimation. The SF block with the filtering operation is constructed in different parts of the SF-CNN to remove the noise from the features containing rain streak information. The GDD block continuously takes the pair of the convolutional operation with the activation function to reduce the dimension of features. Our main contributions are (1) an SF block considering the signal filtering process and effectively removing the useless signals and (2) a procedure of gradually decreasing the dimension of the feature able to learn and reserve the information of features. Experiments on the self-collected dataset, consisting of 9394 raining images with six precipitation intensity levels, demonstrate the proposed approach’s effectiveness against the popular convolutional neural networks. To the best of our knowledge, the self-collected dataset is the largest dataset for monitoring infrared images of precipitation intensity.

## 1. Introduction

The understanding of weather conditions has become more critical, and has been discussed for decades due to the dramatic changes in the global climate, in which precipitation intensity is an important issue. The estimation of the precipitation intensity is the fundamental technology underlying various applications, for example, farming, weather forecasting, and climate simulation. Moreover, the abnormal precipitation intensity can cause disasters, such as floods and droughts, threatening human life and property, and destroying the environment.

Various studies have been dedicated to measuring precipitation intensity, and they can be classified into three categories based on the used data sources: gauge-based [1,2], radar-based [3,4], and satellite-based [5,6] approaches. The rain gauge is the earliest and most widely used device to measure precipitation intensity. The basic concept of a rain gauge is to manually or automatically calculate the rainfall using containers to collect rainwater and estimate the rainfall. Various types of rain gauges have been developed in studies to estimate precipitation intensity, but there have been some drawbacks; for example, the rain gauge must be placed on a flat surface perpendicular to the horizontal plane. Moreover, the rain gauge can only collect the installed local rainfall information. The number of rain gauges needing to be built increases to expand the coverage, which needs a huge budget.

Radar-based approaches use radar to emit radio waves to the sky and receive the radio waves reflected back from various objects. Researchers analyze the reflected radio waves to obtain weather information, such as the moisture content in the air and rainfall probability. The monitoring coverage of radar-based approaches is broader than gauge-based approaches, but the accuracies of the radar-based approaches are related to the reflected waves vulnerable to outside interference, such as the terrain and the reflected waves from various objects.

Satellite-based approaches require various sensors to obtain visibility, infrared, and microwave information for analyzing the precipitation with a large-scale observation. However, the visible and infrared data can only provide information from the top of clouds, which is weakly correlated with rainfall. The microwave emits from the satellite, penetrates the cloud and obtains the information from under the cloud. Therefore, some studies use microwaves to analyze precipitation information. However, a satellite has drawbacks of a low sampling rate and low spatial resolution of visibility, infrared, and microwave.

In addition, surveillance cameras are another kind of sampling equipment which have been widely erected and used in various areas, such as traffic monitoring [7], home care [8], and security monitoring [9]. These studies focus on understanding the scene’s content, including static and dynamic objects. A challenge in analyzing a scene’s contents is related to the weather. In weather-related topics, the rain streaks affect the quality of the monitoring images and distort the interesting objects, reducing the performance of outdoor vision surveillance systems. Therefore, many scholars focus on solving rain streaks by utilizing popular convolutional neural networks in a monitoring image or a surveillance video [10,11]. These methods can detect and remove rain streaks, but do not analyze or provide information on the precipitation intensity.

**Figure 1:**
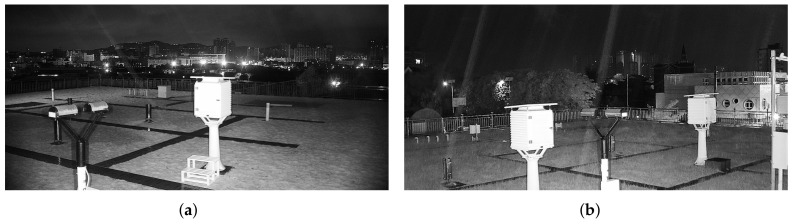
Example of the self-collected precipitation intensity dataset. (**a**) Drizzle, (**b**) moderate rain.

In recent years, many countries have faced the problem of heavy precipitation. Therefore, an efficient approach for estimating precipitation intensity in a city is needed. Figure 1 shows examples of raining images from various weather stations using infrared cameras; Figure 1a,b are images of a drizzle and moderate rain with 0.7 mm and 2.7 mm precipitation, respectively. In Figure 1, rain streaks can be observed in infrared cameras, and that is related to the intensity of precipitation. This study takes infrared surveillance cameras as data sampling devices and designs a new framework—the signal filtering convolutional neural network (SF-CNN)— capable of describing the features of precipitation with signal filtering and dimensional transformation in the infrared image to estimate the precipitation intensity in the city. Our study takes surveillance cameras as data collection devices, providing a high sampling spatial resolution compared to existing methods. Moreover, the SF-CNN achieves more superior results than other popular networks. To the best of our knowledge, no precipitation intensity dataset with surveillance-based infrared images has been created to date. This study is the first to use surveillance-based optical images for precipitation intensity estimation.

The rest of this paper is organized as follows: We present related literature works in Section 2. In Section 3, we introduce the proposed SF-CNN for the precipitation intensity estimation, including the signal filtering block (SF block), the gradually decreasing dimensional block (GDD block), and the entire network structure. Experimental results, a discussion, and conclusions are presented in Section 4, Section 5 and Section 6, respectively.

## 2. Related Works

Precipitation intensity estimation is essential for climate, hydrological, and weather forecasts. The commonly used methods can be classified as the direct rainfall measurement and indirect rainfall estimation.

In the approaches of the direct rainfall measurement, the rain gauges, including the tipping bucket [12,13], weighing rain gauge [14,15], and siphon rain gauge [16], are widely used to measure the precipitation intensity directly. The rain gauge comprises three components: a water receiver (the funnel), a water storage tube (outer tube), and a water container. It uses a water container to collect precipitation and directly measures it by using the methods of weighting and setting thresholds. The precipitation recognition method with the rain gauge sampling is in direct contact with precipitation. The methods consider various containers to collect precipitation and measure the precipitation intensities, playing a critical role in and measuring of precipitation intensity [17]. The rain gauges are the direct and accurate approach to obtaining precipitation [18] and can provide the high-precision measurement of the precipitation intensity at a single sampling point [19]. However, it has the problems of the density of sampling, residual water, and water evaporation.

The rain gauge should be set in an open field to avoid movement and external forces. Therefore, the cost of building a high-density and well-configured rain gauge network is too high. The distribution density of sampling directly affects the accuracy of the precipitation estimation [20] and makes the measured value lose spatial representativeness [21]. Scholars have utilized the interpolation method [22] to fill the lost values in spatial distribution but experience significant errors in large areas because the sampling points are few [23]. In addition, a rain gauge uses a container to receive and estimate the precipitation. It cannot avoid the problems of residual water and water evaporation either using manual observation or automatic measurement. Therefore, more studies consider using approaches that indirectly obtain the precipitation information, such as weather radars, satellite monitoring, and surveillance monitoring, to estimate the precipitation intensity.

Weather radars and satellite monitoring have become essential approaches for large-scale precipitation estimation because the rain gauge has the problems of the limitation of space coverage and the low spatial representation. A weather radar retrieves the rain intensity through reflectivity Z and rainfall intensity R (Z–R relationship), quickly providing real-time precipitation in the study area. Compared with using a rain gauge to measure rainfall intensity, a radar can obtain a higher temporal and spatial resolution and a more comprehensive measurement range by adjusting the emission width and emission frequency. However, the Z–R relationship’s conversion relationship varies with rainfall types [24], and it directly affects the accuracy of the radar. Therefore, determining a reasonable Z–R relationship is a scientific problem when using a weather radar. In addition, precipitation estimation using the radar is susceptible to some factors, such as a non-weather echo, radar beam abnormality, and signal attenuation [25]. These factors can cause differences between the values measured using a radar vs. reality.

In addition, a satellite is a vital sampling device for precipitation intensity measurement, and its spatial coverage is of global scale. Satellite precipitation monitoring mainly consists of infrared (IR) observations from geosynchronous orbit satellites (GEO), passive microwave (PMW) observations from low Earth orbit satellites (LEO), and a combination of the IR and PMW [20]. The IR precipitation estimation is used to associate the properties of the cloud, such as the clou’sd thickness and the top temperature (brightness) of the cloud, to estimate the probability and intensity of precipitation [26]. The lower the cloud top temperature (the brighter the cloud), the stronger the precipitation [27]. However, the relationship between precipitation and the temperature of the cloud top is indirect. Therefore, there exists the estimation error of precipitation intensity. PMW sensors can penetrate clouds and receive microwave signals from hydrometeors compared to infrared signals. PMW sensors can directly measure hydrometeors in the atmosphere [28] to estimate precipitation more accurately [29]. However, satellite images cannot provide direct measurements of precipitation intensity. Moreover, sampling with microwaves has the problems of a low temporal resolution and a large sampling error when processing a short-term rainfall estimation. Therefore, studies have associated the merits of infrared and microwave information with precipitation intensity estimation [5]. Furthermore, scholars associate the remote sensing image, which is provided by a satellite, with precipitation information by assigning precipitation to each pixel to quantify precipitation in the satellite image [30]. Therefore, the quantitative results of precipitation on satellite images depend on the quality of the assignment (classification). Machine learning methods, such as random forests [31] and the convolutional neural network (CNN) [32,33], are effective classification techniques for achieving an image-based precipitation intensity classification. The information provided by the radar and satellite devices is suitable for large-scale precipitation estimation and is brutal regarding measuring the precipitation in small and specific areas. Moreover, the satellite and radar provide precipitation information with large intervals and cannot be used in extreme precipitation monitoring and nowcasting with high timeliness requirements.

In recent years, monitoring cameras have been widely erected, used in various fields, such as traffic, safety, and disaster prevention, and are sampling devices for indirect precipitation intensity estimation. In the early stage, the studies of precipitation intensity estimation using monitoring-based images utilized image processing techniques, such as foreground extraction [34], the morphological component analysis [35], and matrix decomposition [36], to extract rain streaks from the monitoring-based images, used to remove/identify rain streaks from the images. Then, studies use various methods, such as counting [37], neural networks (NN) [38], support vector machines (SVM) [39], and training the identification model for estimating precipitation intensity. These studies utilize the computer vision technique to classify the monitoring-based images for the precipitation intensity estimation.

In addition, the technique of deep convolutional neural networks (DCNNs) has been widely used in various topics of image classification, such as the face [40], vehicle [41], and bird [42], and significantly improved classification accuracy. Scholars associated the stacked denoising auto-encoder (SDAE) [43] with the fully connected structure to construct the PERSIANN-SDAE model and use bispectral information, including infrared and water vapor, to estimate the precipitation intensity [32]. However, the PERSIANN-SDAE model cannot efficiently extract local spatial changes from IR. Therefore, the follower utilizes convolutional neural networks (CNNs) to extract pixel information and information from between pixels from bispectral information for estimating the precipitation intensity [44].

However, the studied topic, precipitation intensity estimation, still has some issues: (1) lacks surveillance-based precipitation intensity dataset which can effectively increase the spatial resolution; (2) the analyzed objects, which are mentioned in the literature, have the apparent shapes compared with the targets (rain streaks) in the precipitation intensity classification; (3) previous studies, which are related to the precipitation intensity estimation, have low temporal and spatial resolutions. Therefore, our study design of the SF-CNN extracts features after applying the signal filtering operation and learns the features with a low dimension for the precipitation intensity estimation. Moreover, our study constructs a surveillance-based infrared images dataset with six precipitation levels.

## 3. Signal Filtering Convolutional Neural Network

This study designed various components to form the signal filtering convolutional neural network (SF-CNN) to construct an effective identification model for the precipitation intensity estimation. The SF-CNN considers the composition of the signal, which is comprised of useful and useless information. Moreover, the SF-CNN effectively decreases the dimension of features to reduce computational costs. This section sequentially introduces the signal filtering block (SF block), the gradually decreasing dimension (GDD block), and the proposed SF-CNN framework.

### 3.1. Signal Filtering Block

**Figure 2:**
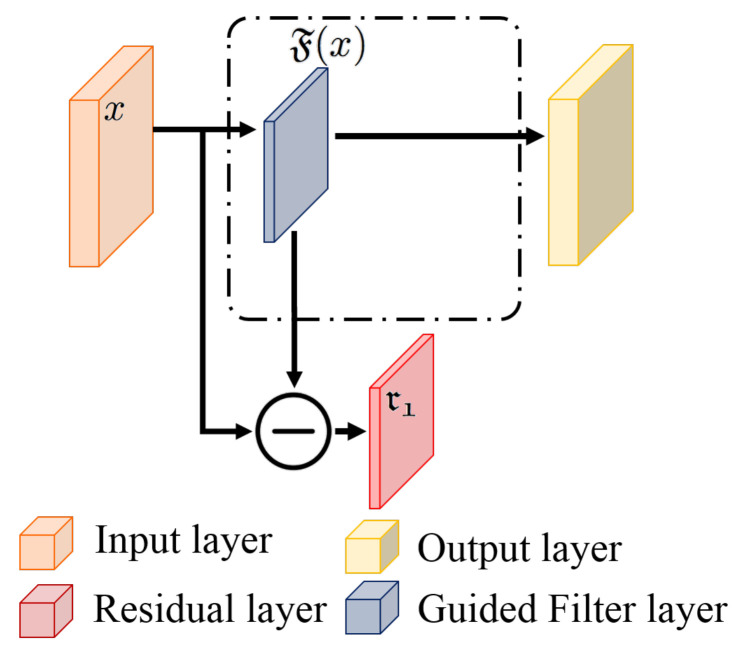
The proposed signal filtering block.

An image can be considered a composite signal containing useless information, such as noise. The noise affects the quality of the image and weakens the characteristics of the interesting objects. This study took the filtering operation to form the signal filtering block (SF block) that could effectively remove the noise from the surveillance-based infrared images and provide the signals without redundant information. The structure of the SF block is shown in Figure 2.

In the SF block, this study took a guided filter, a useful filtering technique, as the signal filtering operation to remove the noise from the analyzed images. The signal filtering procedure could be considered a signal decomposition process that can be restructured into the original signal. Therefore, we took *x* as an input and generated decomposed and residual r signals as outputs for each decomposition procedure (filtering procedure) in which D was also called the filtered signal. Its formula is expressed as the following equation:

(1) x=F(x)+r

where F(.) is the guided filter [45,46] that filters the noise and preserves the characteristics of the gradient from the input *x*, and r is the residue signal of *x* and can be referred to as noise.

This study took the decomposed terms F(x) and D as the inputs and abandoned the last term $r_{n}$ , which was considered as the noise, to construct the signal filtering convolutional neural network.

### 3.2. Gradually Decreasing Dimensional Block

**Figure 3:**
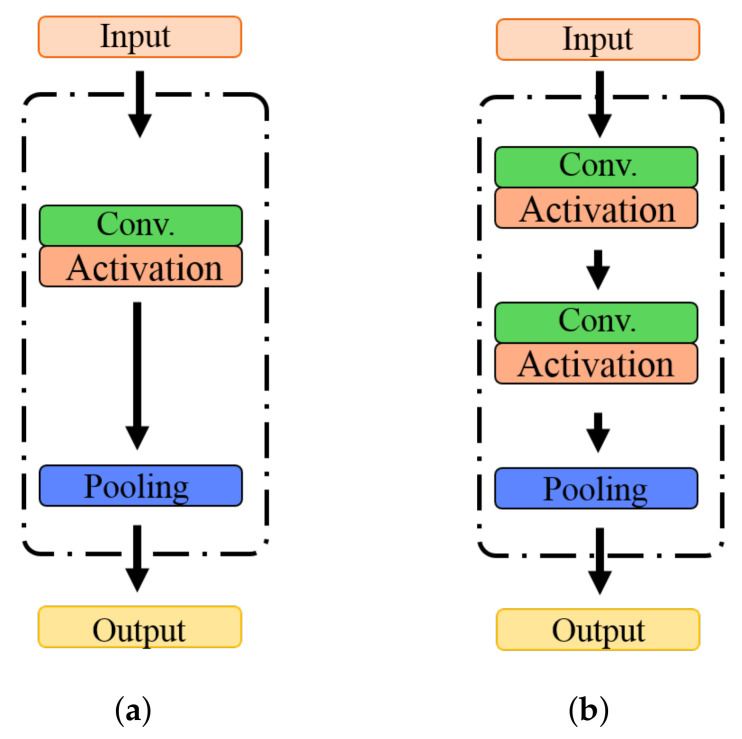
The transformation blocks. (**a**) Standard transformation, (**b**) the proposed gradually decreasing dimensional block (GDD block).

An efficient mechanism to integrate the feature maps from various convolutional layers is crucial in constructing convolution neural networks. The transformation layer is the commonly used component that has one convolutional layer with an activation function and a pooling layer to reduce the dimension of feature maps as shown in Figure 3a. The transformation layer can reduce dimensionality and consider the nonlinearity, but its nonlinearity information is insufficient, and parameters and computational costs are numerous. Therefore, our study designed a gradually decreasing dimensional block (GDD block) with three components, including two convolutional layers with activation functions and one pooling layer, as shown in Figure 3b.

In the GDD block, this study took two convolutional layers with activation functions to gradually decrease the dimensionality of feature maps and be expressed as:

(2) g=pooling(Γ(Γ(s)))

where s is the feature map from the previous layers, Γ is an operator, including the convolutional operation and an activation function, and pooling is the pooling operation. The gradually decreasing dimension can improve the information of nonlinearity by operating two activation functions and effectively reducing the dimensionality of feature maps. Moreover, this study used the max-pooling layer after operating two convolutional layers to decrease the size of feature maps, reducing the computational cost of the network.

### 3.3. Network Structure

**Figure 4:**
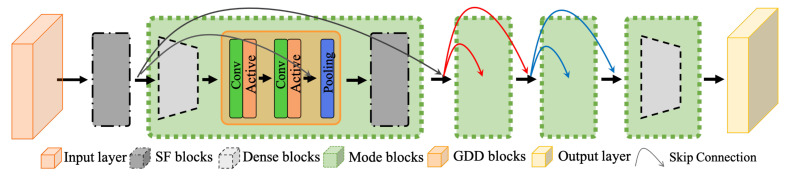
The proposed signal filtering convolutional neural network (SF-CNN).

The proposed signal filtering convolutional neural network (SF-CNN) considered the decomposed signals for precipitation intensity estimation. As shown in Figure 4, it had one input layer, four signal filtering blocks, four mode blocks, and one classification layer.

In the SF-CNN, the input images were resized into 224×224 ; each mode block ( $M_{j}$ ) consisted of two components, including convolutional layers and gradually decreasing dimensional blocks, in which *j* is the number of mode blocks in the SF-CNN. In each mode block, $M_{j}$ firstly operated the convolutional operation for the decomposed terms ( $D_{j}$ ) and generated the set of feature maps $S=\{s_{1},\cdots ,s_{k}\}$ , k∈K ; *K* is the number of feature maps. Each feature map was expressed as:

(3) $$s_{i}=H\left(\sum_{p=1}^{P}D_{j,p}\cdot w_{i}+b_{i}\right)$$

where $D_{j,p}$ is the *j*th decomposed term which has *P* maps; $w_{i}$ and $b_{i}$ are the weight and bias for each $s_{i}$ ; H(.) is the activation function. Then, we applied the GDD block and considered the character of the dense local connectivity for the following layers. The formula of the GDD block was expressed as follows:

(4) $$g_{j}=concat\left(\;\Gamma \left(\Gamma \left(S\right)\right),D_{j}\right)\;$$

where $g_{j}$ is the output of the $M_{j}$ mode block, concat(.) is the concatenation operator which concatenates the previous multiple layers and $\Gamma =H(Conv_{1\times 1}\left(.\right))$ in which $Conv_{1\times 1}$ is the convolutional layer with a 1×1 kernel size. Next, we adopted the pooling layer to reduce the size of feature maps:

(5) $$O_{j}=T\left(g_{j}\right)=pooling\left(g_{j}\right)$$

where $O_{j}=\left\{t_{0},t_{1},\cdots ,t_{d}\right\}$ is the output of the *j*th mode block which was a set with *d* feature maps; pooling is the maximum pooling which operated the 2×2 operator with stride two to reduce the size of the feature maps.

## 4. Experimental Results

This study first presented the self-collected dataset for the precipitation intensity estimation and comparative evaluations in the experiments. Next, the comparison results of the proposed SF-CNN with several popular methods were presented. Finally, this study demonstrated the performance of the proposed SF-CNN without the proposed components to prove the efficiency of these components. All networks were trained using a momentum optimizer, the activation function was ReLU, the batch size was 16 for 400 epochs, and the learning rate was set to 0.0001. The number followed by the method name refers to the network layers.

### 4.1. Experimental Environments and Benchmarks

This study collected the precipitation intensity images from eight weather stations with benchmarks captured by an infrared camera and classified the images into six precipitation intensities according to the grade of precipitation (GB/T 28592-2012). The grade of precipitation and the dataset of precipitation intensity are shown in Table 1. In Table 1, the precipitation intensity was classified as scattered rain, a drizzle, moderate rain, heavy rain, rainstorm, and large rainstorm, based on hourly rainfall. The total number of images was 9394, in which scattered rain, drizzle, and moderate rain accounted for 94.27%. Moreover, this study took 6594 and 2800 images as the training and testing datasets, respectively, which caused the ratio of training and testing images to approximate 7:3, based on the references [47,48].

Moreover, this study took the precision metric and Kappa metric ( κ ) to evaluate the performance of each network. The precision metric was calculated according to the following equation:

(6) $$Overall\;Precision\left(OP\right)=\frac{TP}{TP+FP}\times 100\%$$

where TP and FP are the true and false positives of precipitation intensity. The κ was calculated as follows:

(7) $$\kappa =\frac{p_{0}-p_{e}}{1-p_{e}},\mathrm{where}$$

(8) $$p_{0}=\frac{\sum_{i=0}^{C=6}TP_{i}}{N},p_{e}=\frac{\sum_{i=0}^{C=6}S_{i}\times P_{i}}{N\times N}$$

where *C* is the number of types of precipitation intensity, *N* is the total number of testing images, $TP_{i}$ refers to the images which belong to the *i*th precipitation intensity and are classified as *i*th precipitation intensity, $S_{i}$ is the number of testing images for the *i*th precipitation intensity, and $P_{i}$ is the number of images which were classified as the *i*th precipitation intensity.

### 4.2. Experimental Analysis on Various Networks

This study compare the SF-CNN with eight popular CNN methods, including four classic CNNs—VGG [49], Inception [50,51], the series of ResNets [52], the series of DenseNets [53]—and four novel CNNs—the series of DCNet [54], NTS [55], DCL [56], and HRNet [57]—to evaluate the performance of the proposed SF-CNN.

This study first demonstrated the summarized quantitative comparison results in Table 2. In Table 2, the best results of each benchmark and that of each metric were marked in bold. ResNet-101 and DCNet-101 had the best precision in the benchmark of the large rainstorm; DenseNet-63 and DenseNet-169 had the best precision in the benchmark of the rainstorm; the proposed SF-CNNs had the best results in all the benchmarks. Moreover, the SF-CNN-169 had the best overall precision and κ , and the SF-CNN-63 had fewer parameters. The best overall precision and κ of the SF-CNNs were 2.28% and 0.0360 higher than ResNet-101, the second-best method, respectively. It improved the precision of precipitation intensity estimation, and its performance was superior to the compared methods. The overall precision and κ of SF-CNN-63 were 1.82% and 0.0285 higher than ResNet-101, respectively.

**Figure 5:**
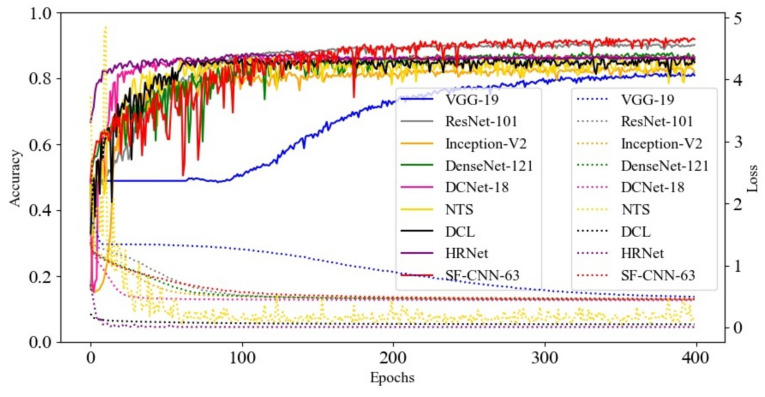
The learning curve of various networks.

In addition, this study presented the learning curve of various networks in Figure 5 to demonstrate the process of training loss and testing accuracy. Notice that this study selected the network which had the best overall precision in its series of networks to form Figure 5, except the proposed SF-CNNs. In Figure 5, this study took the SF-CNN-63 to compare with the other methods because it had the least number of parameters and similarity of precision in the series of SF-CNNs. In Figure 5, the loss value of each method reduced to a lower level, and it did not have the rebound phenomenon. Moreover, the accuracy of each approach gradually increased without the phenomenon of suddenly declining during the training process. Therefore, there was no over-fitting phenomenon in the process of model generation. Furthermore, VGG-19 had the lowest convergence rate and worst classification accuracy; the rest of the compared methods had fast convergence rates, and the increasing accuracy rates were fast at the beginning of training, but their best performance did not exceed the SF-CNN-63 after 300 epochs. Although the proposed SF-CNN-63 had a low convergence rate and unstable accuracy at the beginning of training, its convergence rate became stable and had a good classification accuracy after 300 epochs.

The series of SF-CNNs had the best overall precision, and the κ metric had the best classification performance in all types of precipitation intensities. Moreover, the series of SF-CNNs had the smallest number of parameters compared with the state-of-the-art methods.

### 4.3. Ablation Experimental Analysis of the Proposed Network

The proposed SF-CNN contained the gradually decreasing dimensional block (GDD block) and signal filtering block (SF block). This study modified the proposed SF-CNN-63 and generated the SF-CNN-63-W-GDD and SF-CNN-63-W-SF, in which the SF-CNN-63-W-GDD and SF-CNN-63-W-SF were the SF-CNN-63 without the GDD and SF blocks, respectively, to verify the performance of each proposed block, GDD, and SF blocks. In other words, the SF-CNN-63-W-GDD only contained the SF blocks and was used to verify the performance of SF blocks, and the SF-CNN-63-W-SF only contained the GDD blocks and was used to verify the performance of GDD blocks

The comparison results with the metrics of OP , κ , and *Params* are demonstrated in Table 3. In Table 3, although the GDD and SF blocks were, respectively, removed from the SF-CNN-63, their precision was 4.32% and 0.39% higher than DenseNet-63, respectively. Moreover, their κ was 0.0674 and 0.0039 higher than DenseNet-63, respectively, and their *Params* was less than DenseNet-63. Moreover, the parameters of the SF-CNN-63-W-SF and SF-CNN-63 were the same because the calculation of parameters was related to the neurons (convolutional kernels). The procedure of the SF block was irrelevant to the neurons (convolutional kernels). Although the models of the SF-CNN-63-W-GDD and SF-CNN-63-W-SF had good performance compared to DenseNet-63, the complete model, the SF-CNN-63, had the best performance in each metric.

## 5. Discussion

### 5.1. Analysis of the GDD Block

The gradually decreasing dimension (GDD) block sequentially operated two sets of 1×1 convolutional operations accompanying an activation function. It reduced the dimensionality of feature maps to reduce the number of parameters effectively [50,53]. Moreover, the operation of the gradually decreasing dimension could also achieve the interactive integration of cross-channel information [58], improving the nonlinearity information to improve the expressive ability of the network.

In more detail, the 1×1 convolution operation reduced the number of channels and decreased the computation cost due to the concatenation operation increasing the number of channels. Moreover, the 1×1 convolution operation operated the linear operation to achieve the information combination between channels and reduced the channel dimension. In our study, we sequentially operated two 1×1 convolution operations because the deduction with more channels at once would cause information loss. In addition, this study operated the activation function, which was executed following each convolutional operation to improve the nonlinearity information. The network only constructed with the multi-layer convolution operation could be transferred to a single-layer convolution operation by using a matrix transformation. The activation function executing space mapping with the nonlinear function caused the “multi-layer” of the neural network to have practical meaning and strengthened the learning ability of the model [2,59,60].

**Figure 6:**
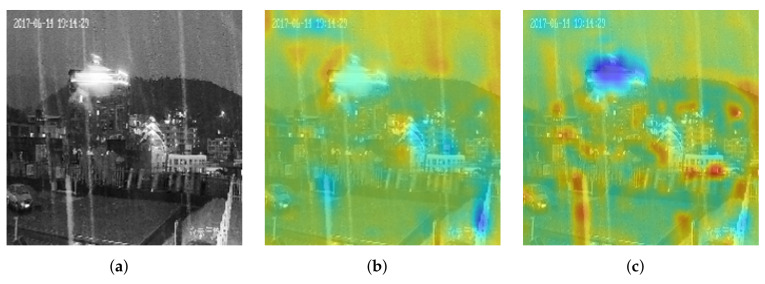
The visualization of skip connection in the second GDD block. (**a**) Original image, (**b**) before, (**c**) after.

In addition, we utilized the skip connection structure to fuse the features in the GDD block, including the features extracted from the SF block and the two 1×1 convolution operations, respectively. The fused features caused the network to continue to focus on rain patterns during the convolution process due to the reuse of the features [53,61], which were filtered by the guided filter and improved the accuracy of precipitation estimation. This study visualized the features’ heatmaps, which were extracted from the GDD block in the second block, as shown in Figure 6.

In Figure 6, Figure 6a–c are the input image, its heatmap before the skip connection operation, and its heatmap after executing the skip connection structure. In Figure 6b,c, the darker the color, the more significant the value. To compare Figure 6b,c, the network focused on the rain pattern after reusing the features filtered by the guided filter. In Figure 6c, the rain streak had higher values than the rest in the input image.

Finally, this study used the max-pooling layer after operating two convolutional layers to decrease the size of feature maps, further reducing the computational cost of the network. The total number of parameters is shown in Table 2. In Table 2, the proposed SF-CNN had the lowest number of parameters in comparison to the compared method with the same depth.

### 5.2. Analysis of the SF Block

In the problem of the precipitation intensity estimation, the noise was an essential issue in the analyzed image that caused the rain streak to be unclear and affected the estimation accuracy in the infrared image. This study took the technique of guided image filtering [46] to minimize noise and background information. The strategies of the guided image filtering were (1) using the mean filter to minimize the noise and background information when the variance was small in the mask area, and (2) maintaining the rain streak (foreground) information that had a large variance.

**Figure 7:**
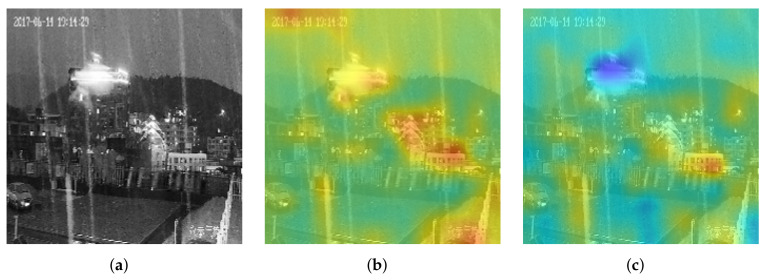
The visualization of the guided filter in the second SF block. (**a**) Original image, (**b**) before, (**c**) after.

This study demonstrated the visualization results to discuss the effects of the SF block, as shown in Figure 7. In Figure 7, Figure 7a–c are the input image, its heatmap before using the guided filter, and its heatmap after executing the guided filter. In Figure 7b, the network payed more attention to the background than to the rain streak before using the guided filter, such as the building with a strong light at the top of the mountain. From Figure 7b,c, the operation of the guided filter could efficiently suppress the background information and retain the rain streak information, resulting in the characteristics of the rain streak to be more prominent.

In the precipitation image, there was more background information than rain streak information, affecting the network by the background during the training process. Therefore, this study added the signal block, which operated the guided filter, before each dense block to ensure the network always focused on the rain streak and avoided the interference of background information during the training process.

### 5.3. Characteristic of the DNN’s Black Box

In traditional machine learning, designers design the image features according to the characteristics of images and construct the classification mechanism based on the mathematical model. Therefore, traditional machine learning approaches are interpretable. The deep convolutional neural network was developed based on the neural network, which is an interpretable mathematical model. Still, it cannot explain why the generated features can efficiently describe the input data. Therefore, many scholars consider that the DNN is a black box technology. In other words, it is difficult to explain its working mechanism for specific reasons for the formation of the features and decision boundaries in the form of mathematical expressions [62]. The relationship between the DNN and interpretability is equivalent to a steam engine and thermodynamics, developing from technological invention to scientific theory. Therefore, the DNN is defined as a “black box”, mainly because there is no primary theoretical basis.

Scholars divide the interpretability of the DNN into two types: post hoc interpretability and intrinsic interpretability [63,64]. The post hoc interpretability interprets the decisions in the actual application [65], in which the visualization method is the widely used approach [66]. The visualization method visually presents the weight of the convolution kernel in the network, the characteristics of the convolution layer, and the object of interest of the model in a visual form. The visualization method can assist researchers in understanding the feature which is extracted from various layers in the DNN. This study adopted the technique of post hoc interpretability (the visualization method) and was associated with the structure of the proposed model to understand the principle of prediction.

### 5.4. Limitation and Outlook

This study designed the signal filtering block by utilizing the guided filter layer to filter out the noise (the residual layer). However, it was possible to retain valuable information in the residual layer. Therefore, we considered the signal decomposition technology [67] to reuse the information from the residual layer. Moreover, the magnitude of the filtered signal would be smaller than the original value. Therefore, these filtered signals should consider the signal enhancement technology, such as the spatial and channel attention in the CNN [68,69].

This study utilized the surveillance-based infrared image captured from eight weather stations, but did not consider the temporal and spatial correlation [70,71]. In the future, we could extend the study to analyze the relationship between sampling points and further study the prediction of the precipitation intensity [72,73].

## 6. Conclusions

This study proposed a new CNN model known as the SF-CNN for precipitation intensity estimation. The concept of the SF-CNN is to consider the decomposed signals using the filtering operation in the network. In the SF-CNN, this study designed a signal filtering block (SF block) and a gradually decreasing dimensional block (GDD block). In the SF block, this study took the guided filter to filter the noise of the feature maps, which was the residual signal. The GDD block used gradually decreasing dimensions to reduce the dimensionality of feature maps. The SF block removed the noise at the beginning of the network and in the entire network procedure. The GDD integrated the information of feature maps, improved the information of nonlinearity, and efficiently reduced the number of parameters.

In the experiments, this study analyzed various network factors for the proposed SF-CNNs and chose the best framework to compare with various popular methods. Comparing the proposed SF-CNNs with various popular methods using the self-collected precipitation dataset, the proposed model exhibited the best overall precision and κ metric, and had the most minor parameters.

## Figures and Tables

**Table 1:** Dataset of precipitation intensity.

Grade of Precipitation (mm/hr)							
Amount	Grade	Scattered Rain (<0.1)	Drizzle (0.1–0.5)	Moderate Rain (1.6–6.9)	Heavy Rain (7.0–14.9)	Rainstorm (15.0–39.9)	Large Rainstorm (40.0–49.9)
Station		(I)	(II)	(III)	(IV)	(V)	(VI)
WS1		209	467	410	96	12	0
WS2		232	518	380	36	48	12
WS3		136	545	389	72	8	0
WS4		101	579	367	78	12	0
WS5		121	651	363	68	12	0
WS6		170	616	339	24	0	0
WS7		224	694	207	24	0	0
WS8		219	504	415	36	0	0
Total		1412	4574	2870	434	92	12

**Table 2:** Quantitative comparison results.

Model	Source	Year	Depth	I (%)	II (%)	III (%)	IV (%)	V (%)	VI (%)	OP (%)	κ	*Params*(M)
VGG	ICLR	2015	19	89.05	86.56	75.99	48.00	60.00	0.00	81.64	0.7121	10.16
Inception-V2	PMLR	2015	32	89.05	88.53	76.57	59.20	80.00	0.00	83.46	0.7411	10.16
ResNet	CVPR	2016	50	92.86	94.08	85.78	56.80	72.00	0.00	89.39	0.8326	23.52
			101	96.19	93.43	87.30	67.20	72.00	**33.33**	90.54	0.8519	42.51
			152	92.38	91.89	82.63	49.60	76.00	0.00	87.00	0.7954	58.16
DenseNet	CVPR	2017	63	93.10	89.70	86.25	50.40	**84.00**	0.00	87.25	0.8010	2.31
			121	93.81	91.02	84.85	56.80	76.00	0.00	87.79	0.8095	6.96
			169	92.38	90.72	82.75	61.60	**84.00**	0.00	87.07	0.7981	12.49
DCNet	CVPR	2018	18	92.38	90.36	83.68	60.80	68.00	0.00	87.00	0.7962	41.93
			101	93.10	91.38	79.60	52.80	56.00	**33.33**	85.93	0.7784	42.58
NTS	ECCV	2018	50	93.81	91.60	84.83	36.80	68.00	0.00	87.07	0.7962	26.25
DCL	CVPR	2019	50	96.90	89.92	79.14	69.60	80.00	0.00	86.57	0.7923	23.50
HRNet	PAMI	2020	50	95.00	90.07	83.57	66.40	72.00	66.67	87.57	0.8081	39.20
SF-CNN	-	2021	63	95.95	95.33	88.81	**76.80**	76.00	**33.33**	92.36	0.8804	**1.99**
			121	**97.14**	94.23	**90.68**	71.20	**84.00**	**33.33**	92.39	0.8814	5.27
			169	96.67	**95.98**	89.04	75.20	80.00	**33.33**	**92.82**	**0.8879**	9.12

Bold data: Highest recognition accuracy of each precipitation intensity.

**Table 3:** The quantitative results without using the proposed blocks.

	SF-CNN-63-W-GDD	SF-CNN-63-W-SF	SF-CNN-63
OP (%)	91.57	87.64	92.36
κ	0.8684	0.8049	0.8804
*Params* (M)	1.85	1.99	1.99

## Data Availability

Data sharing not applicable.
